# Knowledge, attitude, and practice among mothers toward breastfeeding and complementary feeding in community health setting, Malaysia

**DOI:** 10.1016/j.heliyon.2024.e39746

**Published:** 2024-10-26

**Authors:** Halimah Jalil, Mei-Chan Chong, Muhammad Yazid Jalaludin, Li-Ping Wong, Nant Thin Thin Hmwe

**Affiliations:** a Department of Nursing Science, Faculty of Medicine, Universiti Malaya, 50603, Kuala Lumpur, Malaysia; b Dean's Office, Level 5, Faculty of Medicine, Universiti Malaya, 50603, Kuala Lumpur, Malaysia; c Department of Social and Preventive Medicine, Faculty of Medicine, Universiti Malaya, 50603, Kuala Lumpur, Malaysia

**Keywords:** Knowledge, Attitude, Practice, Breastfeeding, Complementary feeding, Community health setting

## Abstract

**Background:**

Breastfeeding for the first six months and complementary feeding until twelve months are crucial for child growth. A mother's knowledge, attitude, and practice (KAP) on infant feeding significantly impact infant development.

**Objective:**

To assess mothers' KAP toward breastfeeding and complementary feeding.

**Methodology:**

A cross-sectional study of 200 mothers with 18- to 24-month-old children at six suburban health clinics in Malaysia. Data were collected via a self-explanatory questionnaire and analyzed using descriptive statistics, chi-square, and Spearman's Rho.

**Results:**

Most mothers had good KAP: 72.5 % had good knowledge, 75.5 % had a positive attitude, and 87 % had good practice. Factors such as maternal age (30–39), multiparity, and vaginal delivery were associated with KAP. Significant positive correlations were found between knowledge and attitude (r = 0.591) and attitude and practice (r = 0.525).

**Conclusions:**

Continued education on breastfeeding and complementary feeding is essential for improving infant feeding practice, and enhancing child development, potentially reducing healthcare costs.

## 1 Introduction

Exclusive breastfeeding (EBF) provides a balanced mix of nutrients, bioactive proteins, ingestible oligosaccharides, signaling system components, bifidogenic bacteria [1], and protection against infections [2]. Additionally, EBF is associated with a lower risk of obesity and diabetes [3,4], asthma [5] and may also have positive effects on cognition [6,7]. The World Health Organization (WHO) has recommended that infants be breastfed within the first hour of birth, exclusively for six months, and that mothers introduce solid foods at six months and continue breastfeeding for up to 2 years [8,9]. However, globally, EBF rates were just 44 % over the period from 2015 to 2020 [9].

In Malaysia, despite the efforts of the government and health professionals, the rate of EBF is at 47.1 %, which is below the global target of 50 %. Additionally, the rate of continued breastfeeding among children aged 20–23 months old is even lower, at 39.4 % [[10], [11], [12]]. This unsatisfactory breastfeeding status could hinder the growth and health of the newborns.

Growth during infancy is greater than at any other age after birth; this includes brain development, which in turn affects mental health [13,14]. Thus, adequate and proper nutrition during infancy and early childhood is essential for ensuring healthy growth and development and can also help reduce the risk of diseases later in life. Although breast milk is very important for infants, it alone cannot provide all the necessary nutrition for a six-month-old infant.

Introducing complementary feeding from six months onwards, in sufficient amounts, at regular intervals, nutritional value, variety, and consistency is paramount to promoting health, supporting growth, and enhancing the child's development [15,16]. Providing infants with a variety of foods is essential to meet their nutritional needs, as well as introducing them to different tastes and textures. A well-rounded diet for infants includes breast milk, cereals, roots and tubers, legumes, nuts and seeds, dairy products, meat products, eggs, as well as vegetables and fruits rich in vitamin A. Providing a diverse diet is important for infants to ensure they receive essential micronutrients such as vitamin A, iron, calcium, thiamine, folate, zinc, vitamin B_6_, and B_12_ [17].

According to the WHO [9], infants 6–8 months old should be fed 2 to 3 times daily, while infants 9–23 months old should be fed 3 or 4 times daily, with 1–2 snacks. Paying attention to the infant's hunger and fullness cues is important. When starting complementary feeding, mothers should begin with 2 or 3 teaspoons and gradually increase the amount. As the infant grows, pureed foods, lumpy foods, and finger foods should be introduced, transitioning to chopped family foods by 12 months of age.

Poor complementary feeding practices can increase the risk of undernutrition, illness, and poor health in adulthood for young children. In 2022, an estimated 149 million children under the age of 5 years suffered from stunting, while 37 million were dealing with overweight or obesity in 2022. Almost half of all deaths among children are linked to undernutrition, which is prevalent mainly in low- and middle-income countries [18]. Hence, early nutritional support, especially breastfeeding, is essential for promoting and maintaining infant and child health.

Various studies have been conducted globally to explore mothers' knowledge, attitude, and behaviors regarding breastfeeding and complementary feeding. However, few studies have focused on breastfeeding and complementary feeding in Malaysia. Therefore, this research seeks to evaluate mothers' knowledge, attitude, and practice regarding breastfeeding and complementary feeding in the community health setting of Gombak District, Malaysia. The study results will be utilized to develop comprehensive educational programs on infant feeding within the country.

The significance of this study lies in its focus on both breastfeeding and complementary feeding, addressing a gap in research that often examines these practices separately in Malaysia. By combining both, the study offers a more comprehensive view of infant nutrition. Conducted in a community setting, it provides practical insights into local knowledge and attitude toward infant feeding. Additionally, the findings contribute fresh data to the literature, reflecting recent changes in knowledge and practices compared to older studies.

## 2 Materials and methods

### 2.1 Study design

The study used a cross-sectional descriptive design in a community health setting.

### 2.2 Setting

The research was conducted in several Maternal and Child Health Clinics (MCHCs) in the Gombak district, Selangor, Malaysia, including Selayang, Gombak Setia, AU2, Taman Ehsan, Sungai Buloh, and Rawang Health Clinics. These clinics were visited by mothers bringing their children for health check-ups and immunizations and to monitor their developmental progress. All mothers who visited the clinics received routine health education from the nurses regarding breastfeeding and complementary feeding during antenatal and postnatal periods. The clinics consistently practised baby-friendly policies, aligning with the Baby-Friendly Hospital Initiative (BFHI) guidelines established by 10.13039/100004423WHO and 10.13039/100006641UNICEF

### 2.3 Participants

The mothers were recruited using convenience sampling. The inclusion criteria were mothers: 1) who had delivered a single, healthy, full-term child, 2) with a child aged between 18 and 24 months, and 3) who were permanent residents in the research area. The exclusion criteria were mothers: 1) who had never breastfed their children, 2) who had not started complementary feeding, and 3) whose baby had been hospitalized or had chronic illness.

### 2.4 Sample size determination

Using the online Raosoft sample size calculator with a 5 % margin of error, 95 % confidence level, a population size of 20,000 (unknown), and a 50 % response distribution, the recommended sample size was 267 [19]. However, the researcher successfully collected data from 200 respondents, aligning with similar studies conducted in Ghana and South Africa [[20], [21], [22]]. The shortfall in sample size was due to incomplete data, logistical challenges, and time constraints.

### 2.5 Research instrument

Data was obtained through the researcher's questionnaire. The researcher used a modified and adapted questionnaire from the Iowa Infant Feeding Attitude Scale [23,24], and previous similar studies [21,22], and it consisted of four sections.

Section A of the questionnaire comprised eight questions regarding mothers' sociodemographic characteristics, including age, ethnicity, education level, household income per month, occupation, number of children, and place of delivery. Section B consisted of 20 questions on mothers' knowledge about infant feeding with optional answers of ‘Yes,’ ‘No,’ or ‘Don't Know.’ The response was converted to a numerical value of 1 for ‘Yes,’ the correct answer, and 0 for both ‘No’, the incorrect answer, and ‘Don't Know.’ The scores were classified into three categories based on the level of knowledge: poor (0–6), moderate (7–13), and good (14–20). Section C contained 20 questions on attitude toward infant feeding, rated using a Likert scale. The Likert scale ranged from 1 = strongly disagree, 2 = disagree, 3 = uncertain, 4 = agree, and 5 = strongly agree. The total score was classified into three levels of attitude: poor (20–46), moderate (47–73), and good (74–100). Section D included 19 questions about infant feeding practice. The Likert scale ranged from 1 = never, 2 = sometimes, and 3 = always. The total scores were dichotomous to two levels of practice: poor (19–37) and good (38–57).

The research instrument was subjected to reliability and validity testing to ensure high-quality data collection. The validity of the questionnaire was established through a panel of experts who explored the theoretical construct [25]. The questionnaire in this study was validated by a nursing lecturer with expertise in community health nursing from the Department of Nursing Sciences and two pediatric lecturers from the Department of Pediatrics at the University Malaya Medical Center. The language expert translated the questionnaire into Malay and back into English to ensure consistency.

A pilot study was conducted to test the research efficiency. The reliability of the test was assessed using the Kuder-Richardson Formula 20 online calculator for knowledge (Section B) and Cronbach's alpha for attitude (Section C) and practice (Section D). The Kuder-Richardson Formula 20 measures the internal consistency reliability of tests using dichotomous (yes/no) questions, and the result was good. Cronbach's alpha was used for the multiple-question Likert scale surveys. The results for Section B were 0.70. The mean was 16.5 with a standard deviation of 4.2, and there were 20 statements. The Cronbach's alpha coefficients for Section C (20 statements) and Section D (19 statements) were 0.90.

### 2.6 Data collection and procedures

Data collection commenced once the researcher had obtained informed consent (oral and written) from the mothers, who had been informed of their right to withdraw from the study at any time. The participants who had agreed to participate were briefed on the purpose of the study and provided instructions before the survey was administered to all consenting mothers using non-random sampling, with an equal proportion of participants chosen from each clinic. Each survey took approximately 20 min to complete. The questionnaires were collected immediately after the participants had completed and sealed them in an envelope. Data collection took place between March and July 2019.

### 2.7 Statistical analyses

The data was analyzed using the SPSS (Statistical Package for the Social Sciences) software, version 25.0. Descriptive statistics, such as frequency, percentage, mean, and standard deviation, were used to measure various aspects of the data. Multiple imputations were used to address missing data in the participants' questionnaires to ensure the findings were robust and representative. Subsequently, the chi-square test assessed the relationship between the mothers' sociodemographic characteristics and their knowledge, attitude, and practice. Additionally, for inferential statistics, Spearman's Rho correlation coefficient was used to examine the relationship between 1) knowledge and attitude and 2) attitude and practice. All tests were two-sided, with a significance level of less than 0.05.

## 3 Results

### 3.1 Respondents’ demographic characteristics

The mean age of the respondents was 31.39 ± 4.95 years. Most of them were of Malay ethnicity, had completed higher education at colleges or universities, had a household income ranging from RM 6001 (US 1400.47) to RM 9000 (US 2100.76) per month, belonged to the professional group, were multiparous mothers, had hospital deliveries, and had spontaneous vaginal delivery. The sociodemographic characteristics of the participating mothers are shown in Table 1.

**Table 1:** Socio-demographic characteristics of respondents (N = 200).

Sociodemographic of Mother's Characteristic	n (%)	Mean ± SD
Age (years)		
20 - 29	71 (35.5)	31.39 ± 4.95
30 - 39	117 (58.5)	
40 and above	12 (6.0)	
Ethnicity		
Malay	107 (53.5)	
Chinese	48 (24.0)	
Indian	34 (17.0)	
Others	11 (5.5)	
Education level		
Primary school	0 (0.0)	
Secondary school	48 (24.0)	
College/University	152 (76.0)	
Household income per month		
≤ RM3000	17 (8.5)	5528 ± 1947.19
RM 3001 - RM 6000	129 (64.5)	
RM 6001 - RM 9000	38 (19.0)	
≥ RM 9001	16 (8.0)	
Occupation		
Professional	97 (48.5)	
Non-professional	43 (21.5)	
Own business	23 (11.5)	
Housewife	37 (18.5)	
Number of children		
Primiparous	82 (41.0)	2.02 ± 1.21
Multiparous	118 (59.0)	
Place of delivery		
Hospital	163 (81.5)	
Maternity Center (private)	37 (18.5)	
Mode of delivery		
Vaginal (spontaneous)	101 (50.5)	
Vaginal (assisted)	9 (4.5)	
Caesarean section	90 (45.0)	

Note: SD, standard deviation.

### 3.2 Respondents’ knowledge regarding breastfeeding and complementary feeding

In Table 2, regarding respondents’ knowledge, approximately two-thirds of the respondents answered correctly that breastfeeding should be initiated within an hour after birth (n = 134, 67 %), colostrum is nutritious for infants and should not be discarded (n = 141, 70.5 %), and complementary feeding should be introduced to the infant after six months (n = 144, 72 %), breastfeeding can continue until the infant age of two years old (n = 148, 74 %). Most of the respondents knew that complementary feeding should begin with refined foods (n = 166, 83 %), followed by mashed foods for infants aged 7–9 months (n = 159, 79.5 %), and then minced or chopped foods for infants 10–12 months (n = 154, 77 %). The majority also understand that complementary food should be given slowly with correct measurement (n = 60, 80 %), understand vegetables help to fight diseases (n = 161, 80.5 %), porridge provides a good source of energy (n = 154, 77 %), and milk/fish/chicken are good for promoting growth (n = 157, 78.5 %).

**Table 2:** Respondent's knowledge regarding infant feeding (N = 200).

Statements	Yes n (%)	No n (%)
Breastfeeding should be initiated within 1 h after childbirth	134 (67)	66 (33)
Initial breast production of yellow water (colostrum) is nutritionally useful for the infant and should not be discarded.	144 (72)	56 (28)
Exclusive breastfeeding for six months is considered long enough∗.	84 (42)	116 (58)
Breastfeeding is continued in infants until the age of two years old.	141 (70.5)	59 (29.5)
Breastfeeding promotes mother-infant bonding.	176 (88)	24 (22)
Breastfeeding prevents infants from getting diarrhoea.	156 (78)	44 (22)
Breastfeeding protects mothers from breast cancer.	142 (71)	58 (29)
Frequent breastfeeding in the early period can help reduce jaundice in infants.	154 (77)	46 (23)
A fully breastfeeding woman is less likely to become pregnant three months after delivery than a formula-feeding woman.	122 (62.5)	78 (39)
Public places provide designated areas for breastfeeding.	125 (62.5)	75 (37.5)
Complementary feeding should be introduced to the infant after six months.	148 (74)	52 (26)
Complementary feeding should begin with refined foods that are smooth and soft to aid digestion.	166 (83)	34 (17)
Complementary feeding should be mashed for infants aged 7–9 months to ensure the thick texture is soft.	159 (79.5)	41 (20.5)
Complementary feeding should be minced or chopped for infants aged 10–12 months for easy chewing.	154 (77)	46 (23)
Breastfeeding should be continued while complementary feeding is given.	142 (71)	58 (29)
According to the correct measurement, infants should be given slowly when complementary food is introduced.	160 (80)	40 (20)
The child stopped breastfeeding at the age of two.	114 (57)	86 (43)
Vegetables help to fight diseases.	161 (80.5)	39 (19.5)
Porridge can provide a good source of energy.	154 (77)	46 (23)
Milk/fish/chicken are all good for promoting growth.	157 (78.5)	43 (21.5)

Note: ∗ Reverse code.

### 3.3 Respondents’ attitude regarding breastfeeding and complementary feeding

In Table 3, concerning respondents' attitude, it was found that most respondents agreed or strongly agreed with the following statements: breastfeeding is more manageable than formula feeding (n = 166, 83 %), breastfeeding should continue even if breast milk is insufficient (n = 169, 83 %), breast milk is not sufficient for an infant after six months (n = 143, 71.5 %), it is crucial to ensure that the child does not consume too many sugary foods (n = 176, 88 %), it is essential to ensure that the child does not consume excessive high-fat foods (n = 169, 84.5 %), if the parents do not guide or regulate their child's eating habits, the child will overeat their favorite foods (n = 152, 76 %), and if the parents do not guide or regulate their child's eating habits, the child will eat much less than they should (n = 144, 72 %).

**Table 3:** Respondents’ attitude regarding infant feeding (N = 200).

Statements	SD n (%)	D n (%)	U n (%)	A n (%)	SA n (%)
I decided to breastfeed my child before getting pregnant.	4 (2)	16 (8)	18 (9)	53 (26.5)	109 (54.5)
Breastfeeding does not affect the care of other family members and the marital relationship.	0 (0)	8 (4)	29 (14.5)	86 (43)	77 (38.5)
Breastfeeding prevents overweight.	7 3.5)	18 (9)	33 (16.5)	79 (39.5)	63 (31.5)
Breastfeeding is a good way to decrease family expenses.	0 (0)	15 (7.5)	5 (2.5)	88 (44)	92 (46)
Breastfeeding is more convenient than formula feeding.	0 (0)	23 (11.5)	11 (5.5)	67 (33.5)	99 (49.5)
Breast milk alone is not enough for the infant after six months.	6 (3)	23 (11.5)	28 (14)	72 (36)	71 (35.5)
Expressed breastfeeding is a better choice if the mother plans to go out to work.	0 (0)	10 (5)	16 (8)	83 (41.5)	91 (45.5)
My husband, parents, and friends encourage breastfeeding overfeeding infant formula.	3 (1.5)	21 (10.5)	20 (13.5)	83 (41.5)	73 (36.5)
I feel confident about breastfeeding in public places.	10 (5)	37 (18.5)	27 (13.5)	79 (39.5)	47 (23.5)
I am comfortable breastfeeding my infant.	0 (0)	15 (7.5)	12 (6)	86 (43)	87 (43.5)
Fathers will not feel left out if a mother breastfeeds.	0 (0)	9 (4.5)	29 (14.5)	81 (40.5)	81 (40.5)
Feeding the child with formula milk before the first breastfeed is not advisable.	2 (1)	23 (11.5)	30 (15)	60 (30)	85 (42.5
Maternity leave of 3 months is very short for successful breastfeeding.	2 (1)	50 (25)	22 (11)	59 (29.5)	67 (33.5)
I felt I had a sufficient breast milk supply, and my child was getting enough.	0 (0)	44 (22)	28 (14)	61 (30.5)	67 (33.5)
Mothers who formula feed miss one of the great joys of motherhood.	2 (3.5)	38 (19)	33 (16.5)	60 (30)	62 (31)
Even if the amount of breast milk produced is little, it is recommended to continue breastfeeding.	0 (0)	16 (8)	18 (9)	96 (48)	70 (35)
I need to ensure my child doesn't consume too much sweet food.	0 (0)	6 (3)	18 (9)	89 (44.5)	87 (43.5)
I need to ensure that my child does not consume excessive high-fat foods.	0 (0)	10 (5)	21 (10.5)	80 (40)	89 (44.5)
If I did not guide or regulate my child's eating habits, she would eat too many of her favorite foods.	2 (1)	12 (6)	34 (17)	81 (40.5)	71 (35.5)
If I did not guide or regulate my child's eating habits, she would eat much less than she should.	2 (1)	24 (12)	30 (15)	83 (41.5)	61 (30.5)

Note: SD=Strongly Disagree, D = Disagree, U=Unsure, A + Agree, SA=Strongly Agree.

### 3.4 Respondents’ practice regarding breastfeeding and complementary feeding

In Table 4, it was found that from the respondent's practice that almost all (n = 184, 92 %) breastfed their infants and fed them whenever they were hungry (n = 178, 89 %). Around one-third of the respondents (n = 73, 36.5 %) avoided formula feeding when their infants were between 0 and 6 months old, and of those, 90 (45 %) managed to breastfeed their infants during that time exclusively. After the age of 6 months, 107 (53.5 %) respondents introduced complementary feeding, giving it to their children three times a day. Additionally, 102 (51 %) of respondents gave their child food with carbohydrates twice daily, and 111 (55.5 %) included protein-rich food such as fish, chicken, egg, or bean curd in carbohydrate preparation. Furthermore, 115 (57.5 %) of respondents added vegetables like carrot, green mustard leaf, or spinach into the carbohydrate preparation, while over half (n = 117, 58.5 %) gave their child fruits such as papaya, banana, or orange.

**Table 4:** Respondent's practice regarding infant feeding (N = 200).

Statements	N n (%)	S n (%)	A n (%)
Have you ever breastfed your infant?	16 (8)	74 (37)	110 (55)
Are you still breastfeeding your child?	81 (40.5)	55 (27.5)	64 (32)
Did you breastfeed your child within the first hour of birth?	71 (35.5)	23 (11.5)	106 (53)
Do your family members or friends encourage you to breastfeed your child?	13 (6.5)	52 (26)	135 (67.5)
Do you breastfeed your child in a public place?	59 (29.5)	48 (24)	93 (46.5)
Do you feel comfortable breastfeeding, such as in a public place?	80 (40)	40 (20)	80 (40)
Do you face ease in breastfeeding, such as a good flow of breast milk?	17 (8.5)	59 (29.5)	124 (62)
Did you avoid feeding infant formula to your child when he/she was between 0 and 6 months old?	71 (35.5)	56 (28)	73 (36.5)
Did you give only breast milk without mixing formula milk when your child was aged between 0 and 6 months?	58 (29)	52 (26)	90 (45)
Do you breastfeed your child whenever your child makes sucking noises or cry?	22 (11)	56 (28)	122 (61)
Did you give complementary feeding to your child at 4 months?	64 (32)	44 (22)	92 (46)
Did you give your child complementary feeding after the age of 6 months?	50 (25)	35 (17.5)	115 (57.5)
Do you feed your child with complementary feeding three times a day?	3 (16)	61 (30.5)	107 (53.5)
Do you feed your child food with carbohydrates twice a day?	23 (11.5)	75 (37.5)	102 (51)
Do you mix food with protein when preparing carbohydrates, such as fish, chicken, egg, or bean curd?	31 (15.5)	58 (29)	111 (55.5)
Do you mix vegetables such as carrot/green mustard/spinach into carbohydrate preparation?	29 (14.5)	56 (28)	115 (57.5)
Do you give your child fruits such as papaya/banana/orange?	25 (12.5)	58 (29)	117 (58.5)
Do you give your child other food such as Farley's biscuit/Marrie biscuit/finger foods?	50 (25)	61 (30.5)	89 (44.5)
Do you warm up the food before giving it to your child?	32 (16)	61 (30.5)	107 (53.5)

Note: N = Never, S = Sometimes, A = Always.

### 3.5 Respondents’ knowledge, attitude, and practice levels

In Table 5, most respondents (n = 145, 72.5 %) had good knowledge of infant feeding, with a score range of 14–20 and a mean score of 14.46 ± 5.32. Three-quarters of the respondents (n = 151, 75.5 %) had a positive attitude toward infant feeding, with a mean score of 80.58 ± 14.07. Additionally, an overwhelming majority of the respondents (n = 174, 87 %) reported good practices in feeding their infants, with an average score of 43.64 ± 10.26.

**Table 5:** Knowledge, attitude, and practice levels of respondents (N = 200).

Variable	Score	Frequency	Percentage	Mean ± SD
		(n)	(%)	
Knowledge				
Poor	0–6	16	8.0	14.46 ± 5.32
Moderate	7–13	39	19.5	
Good	14–20	145	72.5	
Attitude Score				
Poor	20–46	4	2.0	80.58 ± 14.07
Moderate	47–73	45	22.5	
Good	74–100	151	75.5	
Practice Score				
Poor	19–37	26	13.0	43.64 ± 10.26
Good	38–57	174	87.0	

Note: SD, standard deviation.

### 3.6 Associations between respondents’ sociodemographic characteristics and knowledge

Table 6 shows that the mother's breastfeeding knowledge was significantly associated with age (*P* < 0.001), education level (*P* = 0.002), household income (*P* = 0.012), occupation (*P* = 0.004), number of children (*P* < 0.001), place of delivery (*P* < 0.001), and mode of delivery (*P* < 0.001). No significant association was found between breastfeeding knowledge and ethnicity (*P* = 0.242).

**Table 6:** Association between sociodemographic characteristics and knowledge (N = 200).

Characteristic	Knowledge level			Chi-square		
	Poor	Moderate	Good	X^2^	df	P-value
	n (%)	n (%)	n (%)			
Age group (years)						
20 - 29	10 (14.1)	23 (32.4)	38 (53.5)	20.11#	4	0.001∗
30 - 39	6 (5.1)	16 (13.7)	95 (81.2)			
40 and above	0 (0.0)	0 (0.0)	12 (100.0)			
Ethnicity						
Malay	9 (8.4)	17 (15.9)	81 (75.7)	7.49#	6	0.242
Chinese	2 (4.2)	10 (20.8)	36 (75.0)			
Indian	3 (8.8)	11 (32.4)	20 (58.8)			
Others	2 (18.2)	1 (9.1)	8 (72.7)			
Education level						
Secondary school	3 (6.2)	18 (37.5)	27 (56.2)	13.03	2	0.002∗
College/University	13 (8.6)	21 (13.8)	118 (77.6)			
Household income per month						
≤ RM3000	0 (0.0)	5 (29.4)	12 (70.6)	14.87#	6	0.012∗
RM 3001 - RM 6000	16 (12.4)	14 (18.6)	89 (69.0)			
RM 6001 - RM 9000	0 (0.0)	10 (26.3)	28 (73.7)			
≥ RM 9001	0 (0.0)	0 (0.0)	16 (100.0)			
Occupation						
Professional	13 (13.4)	11 (11.3)	73 (75.5)	18.06#	6	0.004∗
Non-professional	1 (2.3)	15 (34.9)	27 (62.8)			
Own business	2 (8.7)	6 (26.1)	15 (65.2)			
Housewife	0 (0.0)	7 (18.9)	30 (81.1)			
Parity						
Primiparous	16 (19.5)	33 (40.3)	33 (40.2)	73.64	2	0.000∗
Multiparous	0 (0.0)	6 (5.1)	112 (94.9)			
Place of delivery						
Hospital	9 (5.5)	27 (16.6)	127 (77.9)	14.22	2	0.001∗
Maternity center (private)	7 (18.9)	12 (32.4)	18 (48.6)			
Mode of delivery						
Vaginal (spontaneous)	0 (0.0)	15 (14.7)	87 (85.3)	30.88#	4	0.001∗
Vaginal (assisted)	2 (22.2)	0 (0.0)	7 (77.8)			
Caesarean section	38 (15.7)	24 (27.0)	51 (57.3)			

Note: #, Fisher's exact test, df, degrees of freedom, ∗, Significant <0.05.

### 3.7 Associations between respondents’ sociodemographic characteristics and attitude

Table 7 shows that a mother's attitude towards the feeding of her infant was significantly associated with the mother's age (*P* < 0.001), occupation (*P* = 0.016), number of children the mother had (*P* < 0.001), place of delivery (*P* = 0.003), and mode of delivery (*P* = 0.001). No significant association was found between mothers' attitude toward infant feeding and their ethnicity (*P* = 0.885), education level (*P* = 0.192), or household income (*P* = 0.071).

**Table 7:** Association between socio-demographic characteristics and attitudes (N = 200).

Characteristic	Attitude level			Chi-square		
	Poor	Moderate	Good	X^2^	df	P -value
	n (%)	n (%)	n (%)			
Age (years)						
20 - 29	4 (5.6)	30 (42.3)	37 (52.1)	34.37#	4	0.001∗
30 - 39	0 (0.0)	12 (10.3)	105 (89.7)			
40 and above	0 (0.0)	3 (25.0)	9 (75.0)			
Ethnicity						
Malay	3 (2.8)	23 (21.5)	81 (75.7)	2.50#	6	0.885
Chinese	1 (2.1)	9 (18.8)	38 (79.2)			
Indian	0 (0.0)	10 (29.4)	24 (70.6)			
Others	0 (0.0)	3 (27.3)	8 (72.7)			
Education level						
Secondary school	1 (2.1)	15 (31.2)	32 (66.7)	2.99#	2	0.192
College/University	3 (2.0)	30 (19.7)	119 (78.3)			
Household income per month						
≤ RM3000	0 (0.0)	0 (0.0)	17 (100.0)	10.45#	6	0.071
RM 3001 - RM 6000	4 (3.1)	36 (27.9	89 (69.0)			
RM 6001 - RM 9000	0 (0.0)	6 (15.8)	32 (84.2)			
≥ RM 9001	0 (0.0)	3 (18.8)	13 (81.2)			
Occupation						
Professional	0 (0.0)	25 (25.8)	72 (74.2)	13.58#	6	0.016∗
Non-professional	4 (9.3)	7 (16.3)	32 (74.4)			
Own business	0 (0.0)	8 (34.8)	15 (65.2)			
Housewife	0 (0.0)	5 (13.5)	32 (86.5)			
Parity						
Primiparous	4 (4.9)	35 (42.7)	43 (52.4)	40.36#	2	0.001∗
Multiparous	0 (0.0)	10 (8.5)	108 (91.5)			
Place of delivery						
Hospital	3 (1.8)	29 (17.8)	131 (80.4)	10.95#	2	0.003∗
Maternity center (private)	1 (2.7)	16 (43.2)	20 (54.1)			
Mode of delivery						
Vaginal (spontaneous)	0 (0.0)	13 (12.7)	89 (87.3)	17.63#	4	0.001∗
Vaginal (assisted)	0 (0.0)	3 (33.3)	6 (66.7)			
Caesarean section	4 (4.5)	29 (32.6)	56 (62.9)			

Note: #, Fisher's exact test, df, degrees of freedom, ∗, Significant <0.05.

### 3.8 Associations between respondents’ sociodemographic characteristics and practices

Table 8 shows that mothers' infant-feeding practices were significantly associated with their age (*P* = 0.003), number of children the mothers had (*P* < 0.001), and mode of delivery (*P* = 0.004). No significant association was found between infant feeding practices and mother's ethnicity (*P* = 0.495), education level (*P* = 0.460), household income (*P* = 0.095), occupation (*P* = 0.630), or place of delivery (*P* = 0.103).

**Table 8:** Association between socio-demographic characteristics and practice (N = 200).

Characteristic	Practice level		Chi-square		
	Poor	Good	X^2^	df	P -value
	n (%)	n (%)			
Age (years)					
20 - 29	23 (32.4)	48 (67.6)	12.22	2	0.003∗
30 - 39	21 (17.9)	96 (82.1)			
40 and above	0 (0.0)	12 (100.0)			
Ethnicity					
Malay	16 (15.0)	91 (85.0)	2.24#	3	0.495
Chinese	11 (22.9)	37 (77.1)			
Indian	15 (44.1)	19 (55.9)			
Others	2 (18.2)	9 (81.8)			
Education level					
Secondary school	13 (27.1)	35 (72.9)	0.75	1	0.460
College/University	31 (20.4)	121 (79.6)			
Household income per month					
≤ RM3000	2 (11.8)	15 (88.2)	5.90#	3	0.095
RM 3001 - RM 6000	31 (24.0)	98 (76.0)			
RM 6001 - RM 9000	9 (23.7)	29 (76.3)			
≥ RM 9001	2 (12.5)	14 (87.5)			
Occupation					
Professional	18 (18.6)	79 (81.4)	1.78#	3	0.630
Non-professional	12 (27.9)	31 (72.1)			
Own business	7 (30.4)	16 (69.6)			
Housewife	7 (18.9)	30 (81.1)			
Parity					
Primiparous	38 (46.3)	44 (53.7)	43.00	1	0.001∗
Multiparous	6 (5.1)	112 (94.9)			
Place of delivery					
Hospital	32 (19.6)	131 (80.4)	2.98	1	0.103
Maternity center (private)	12 (32.4)	25 (67.6)			
Mode of delivery					
Vaginal (spontaneous)	12 (11.8)	90 (88.2)	12.07	2	0.004∗
Vaginal (assisted)	2 (22.2)	7 (77.8)			
Caesarean section	30 (33.7)	59 (66.3)			

Note: #, Fisher's exact test, df, degrees of freedom, ∗, Significant <0.05.

### 3.9 Correlations between respondents’ knowledge, attitude, and practice

Table 9 shows a positive correlation between knowledge, attitude, and practice. This means that an increase in knowledge was positively correlated with attitude (r = 0.591, *P* < 0.001), and attitude is positively associated with practice (r = 0.525, *P* < 0.001).

**Table 9:** Correlation between the knowledge, attitude, and practices of the respondents toward infant feeding (N = 200).

		Attitude	Practice
Spearman's Rho	Knowledge	0.591∗∗	–
	Attitude	–	0.525∗∗

Note: ∗∗Correlation is significant at the 0.01 level (2-tailed).

## 4 Discussion

The research evaluated mothers' knowledge, attitude, and practice regarding infant breastfeeding and complementary feeding within a number of community health settings in Malaysia. Notably, the mothers in this study displayed a high level of knowledge, positive attitude, and appropriate practice about infant feeding. It also found a significant link between demographic characteristics and the mothers' knowledge, attitude, and practice. Furthermore, a positive correlation was observed between knowledge and attitude, as well as attitude and practice. The positive findings on breastfeeding knowledge, attitude, and practice in this study align with similar research conducted in other parts of Malaysia [28,29]. However, there remains a gap in studies addressing complementary feeding, or the combination of both breastfeeding and complementary feeding, within the Malaysian context.

In Indonesia, according to WHO recommendations, the practice of infant and young child feeding (IYCF) is still low, starting with early breastfeeding initiation, EBF, and complementary feeding [26]. There is an urgent need for sustainable programs that educate and assist pregnant women in IYCF practices. The sustainable programs could include regular antenatal classes, community-based health education initiatives, and integrating IYCF education into existing healthcare services. This emphasis on sustainability will impress upon pregnant women the importance and urgency of the issue. Another study conducted in Indonesia on complementary feeding revealed a low prevalence of minimum dietary diversity and minimum acceptable diet among children aged 6–24 months [27]. Enhancing infant and IYCF practices requires improved healthcare services, better economic conditions, and higher educational attainment among mothers.

In a study conducted in Thailand by Kittisakmontri et al. [28], it was found that family members other than mothers were less likely to understand the importance of complementary feeding for child growth. They also had misconceptions about the benefits of animal-based protein and lacked confidence in their ability to feed the child properly. While most families followed feeding recommendations, undesirable practices such as delayed introduction of certain foods and offering food as a reward were also observed. The researchers suggested the need for nutritional education for all caregivers involved in complementary feeding.

In a study conducted in Bangladesh [29], it was found that 83.5 % of mothers had good knowledge about IYCF. Almost all participants agreed on the importance of breastfeeding and complementary feeding, but only half practiced it. The study also revealed a significant relationship between a mother's educational background and her knowledge, attitude, and practices regarding IYCF. Similarly, in Nepal [30], the study showed that people were highly knowledgeable about EBF but had little understanding of complementary feeding. The research also identified a gap between knowledge and practice regarding the appropriate age for starting complementary feeding, preparing complementary food, and related practices.

In Turkey [31], parents lacked sufficient knowledge about breastfeeding and human milk. These mothers needed support to continue EBF for the first six months and to provide appropriate complementary food during the weaning feeding. In a study conducted in Turkey [32], although most mothers expressed a positive attitude toward breastfeeding, the proportion exclusively breastfeeding for the first six months was low, as measured by the Iowa Infant Feeding Attitude Scale. Half of the mothers introduced complementary feeding between 4 and 6 months. The study also found that as mothers' education and income levels increased, so did their positive attitudes toward breastfeeding.

In a study conducted in Western Ethiopia [32], 88.9 % of the mothers exhibited good knowledge, 78.2 % had a positive attitude, and 78.2 % demonstrated good practice of IYCF recommendations. In addition, the mother's age, level of education, location of the delivery, father's educational background, his involvement and support, previous knowledge about IYCF, conversations with the husband/partner about IYCF, and attendance at antenatal clinics were found to be significantly linked to the mother's understanding of IYCF recommendations. The researchers suggested implementing behavior change communication intervention strategies to support IYCF in mothers. It also would help to bridge the gap between knowledge and actual practices.

A study conducted in Southwest Ethiopia [33] found that half of the mothers had a high level of knowledge about complementary feeding. However, their attitude and self-efficacy toward complementary feeding were low. The study identified maternal educational status, the number of antenatal clinic (ANC) visits, and the information received about complementary feeding as predictors of complementary feeding knowledge. Additionally, the child's sex and the number of ANC visits were identified as predictors of a mother's attitude toward complementary feeding. Moreover, the size of the family and the household's food security status predicted self-efficacy in implementing complementary feeding. Based on these findings, it was suggested that nutrition intervention strategies are necessary to enhance maternal knowledge, attitude, and self-efficacy toward optimum complementary feeding. In another study by Abiyu and Belachew [34], mothers' knowledge and attitude toward optimal complementary feeding were not very good. Interventions that improve behavior change in terms of optimal complementary feeding are important, focusing on age-specific meal frequency diversification and feeding during and after an illness. Additionally, addressing the negative impact of bottle feeding in the community is necessary.

Mothers in the Najran Region of Saudi Arabia [35] had limited knowledge about the weaning diet and tended to follow local customs. Underfeeding infants compared to the standard four times daily was significantly associated with poorer knowledge scores. The study recommended improving communication channels between physicians and mothers through targeted educational programs and emphasized the importance of this recommendation. Their study suggested that future knowledge about weaning should focus on its association with traditional weaning methods and bottle feeding.

On the other hand, a study in rural areas of Kenya [36] showed that mothers in Nairobi and Machakos scored well on good knowledge of breastfeeding scores (6.3/9 and 5.9/9, respectively). However, the rates of complementary feeding in both areas were poor (7.5/14 in Nairobi and 7.1/14 in Machakos). The researchers recommended that future interventions prioritize improving dietary diversity. In another study conducted in Kenya [37] in a pastoral community, the researchers investigated maternal knowledge of child complementary feeding practices and their impact on the nutritional status of children aged 6–23 months. The study revealed that most mothers/caregivers knew children should be encouraged to feed and should be fed more frequently during and after illness. The timing of complementary feeding and the child's dietary diversity were identified as significant predictors of wasting among the children.

In Ghana [38], mothers' knowledge levels about complementary feeding are inadequate despite their generally positive attitude towards child feeding recommendations. Mothers should be informed about feeding recommendations for infants and young children, and fathers should have sufficient income to support a healthy diet. Nutrition education should enhance mothers' understanding of feeding recommendations for infants and young children and help them overcome obstacles to providing their children with nutritious diets. In another study conducted in Ghana [39], no significant association was found between the mother's knowledge of child nutrition and the nutritional status of children. However, there was a noticeable decrease in wasting with increased nutritional knowledge. The prevalence of malnutrition among the children in the study was lower than the national average. Still, underweight rates were significantly higher in children with mothers or caregivers who were farmers. Therefore, the researchers recommended promoting appropriate nutrition education on IYCF practices during antenatal care and child welfare clinic services within these communities. Additionally, targeted family planning services for teenage girls should be implemented to prevent adolescent pregnancies, as children born to teenage mothers are more likely to experience malnutrition.

A study conducted in South Africa [40], revealed that despite mothers having good knowledge regarding feeding their infants, their actual feeding practices often did not reflect this knowledge. The research also found significant associations between mothers’ feeding practices and the nutritional status of their children. In another South African study [41], many caregivers were aware that breastfeeding should commence immediately after birth which would provide protection against diseases. They also understood that solid food should be introduced at six months. Nonetheless, there remained a need to enhance education on IYCF, particularly on exclusive breastfeeding.

In a study conducted in India [42], the following rates were found: EBF at 58.7 %, early initiation of breastfeeding at 43.8 %, timely introduction of complementary feeding at 43.3 %, minimum dietary diversity at 16.6 %, minimum meal frequency at 27.4 %, and minimum acceptable diet at 6.8 %. The study identified various factors associated with breastfeeding and complementary feeding practices, including maternal education, mode of delivery, frequency of antenatal care (ANC) clinic visits, geographical region, child's age, marital status, and household wealth. Notably, the study revealed that IYCF practices among adolescent mothers were generally suboptimal, except for breastfeeding.

A scoping review [43] highlighted a persistent global disparity in knowledge, attitude, and complementary feeding practice. The review included studies conducted between 2014 and 2022 and focused on Southeast Asia (Indonesia, Bangladesh, and Thailand), West Asia (Saudi Arabia), South Asia (Nepal, India, and Pakistan), Africa (Ghana, Burkina Faso, and Ethiopia), and Europe (Kosovo). Despite regional variations, the common challenges identified suggested the need for universally applicable strategies that can be adapted to local contexts. Addressing these gaps through comprehensive education, supportive policies, and economic interventions can significantly improve complementary feeding practices worldwide.

Despite global improvement in 57 low- and middle-income countries over the past decade (2010–2018), there is still a need to catch up to WHO feeding recommendations [44]. Breastfeeding practices vary significantly across WHO regions, with the Eastern Mediterranean, some European areas, and even upper-middle-income countries facing challenges in meeting targets. Continuing efforts are crucial to achieving the 2025 global breastfeeding target. Further training and educational programs on breastfeeding and complementary training could promote the knowledge, attitude, and behavior of the mothers [45].

The researcher's study has some limitations. First, the study was conducted in community health clinics in a single district, meaning the results may not apply to all mothers with infants in Malaysia. Future studies should be performed at multiple districts to ensure more representative findings. Second, the study was conducted near the capital city of Malaysia, and only suburban residents were included. Mothers from rural areas should be recruited for future studies to make the study more inclusive. Finally, participants may not accurately remember past behaviors, experiences, or exposures, leading to inaccurate or incomplete data.

## 5 Conclusion

Overall, the mothers in this study demonstrated good knowledge, attitudes, and practice (KAP) regarding infant feeding, showing a positive correlation between these factors. Healthcare providers must continue educating mothers on the importance of breastfeeding and complementary feeding. Expanding research to a broader population would further illuminate baby-feeding practices among Malaysian mothers.

Strengthening knowledge about breastfeeding and complementary feeding in community health settings is vital for sustaining these practices, which lead to improved child development and reduced healthcare costs. This can result in economic benefits for families and the country. Therefore, healthcare providers, especially nurses and medical professionals, must prioritize ongoing education for mothers, ensuring that infants receive proper nutrition as Malaysia's Ministry of Health and the World Health Organization recommends.

## CRediT authorship contribution statement

**Halimah Jalil:** Writing – review & editing, Writing – original draft, Validation, Resources, Project administration, Methodology, Investigation, Formal analysis, Data curation, Conceptualization. **Mei-Chan Chong:** Writing – review & editing, Supervision, Project administration, Methodology, Conceptualization. **Muhammad Yazid Jalaludin:** Writing – review & editing, Supervision, Formal analysis. **Li-Ping Wong:** Writing – review & editing, Supervision, Formal analysis. **Nant Thin Thin Hmwe:** Writing – review & editing, Formal analysis.

## Ethical approval

This study was approved by the ethical and institutional review board committee for human investigation at the University Malaya Medical Centre (UMMC) (Number: 22168).

## Data availability statement

The article includes all the relevant data for the study.

## Funding

This research did not receive any specific grant from any funding agency in the public, commercial, or not-for-profit sectors.

## Declaration of competing interest

The authors declare that they have no known competing financial interests or personal relationships that could have appeared to influence the work reported in this paper.
